# The Effect of Obstructive Sleep Apnea on Venous Thromboembolism Risk in Patients Undergoing Total Joint Arthroplasty

**DOI:** 10.5435/JAAOSGlobal-D-21-00248

**Published:** 2022-04-20

**Authors:** Alex Tang, Vinay K. Aggarwal, Richard S. Yoon, Frank A. Liporace, Ran Schwarzkopf

**Affiliations:** 1 From the Department of Orthopedic Surgery, Jersey City Medical Center-RWJBarnabas Health, Jersey City, NJ (Dr. Tang, Dr. Yoon, and Dr. Liporace), and the Department of Orthopedic Surgery, NYU Langone Health, New York, NY (Dr. Aggarwal, and Dr. Schwarzkopf).

## Abstract

Obstructive sleep apnea (OSA) is a known risk factor for venous thromboembolism (VTE), defined as pulmonary embolism (PE) or deep vein thrombosis (DVT); however, little is known about its effect on VTE rates after total joint arthroplasty (TJA). This study sought to determine whether patients with OSA who undergo TJA are at greater risk for developing VTE versus those without OSA.

**Methods:**

A retrospective analysis was conducted on 12,963 consecutive primary TJA patients at a single institution from 2016 to 2019. Patient demographic data were collected through query of the electronic medical record, and patients with a previous history of OSA and VTE within a 90-day postoperative period were captured using the International Classification of Disease, 10th revision diagnosis and procedure codes.

**Results:**

Nine hundred thirty-five patients with OSA were identified. PE (0.6% versus 0.24%, *P* = 0.023) and DVT (0.1% versus 0.04%, *P* = 0.37) rates were greater for patients with OSA. A multivariate logistic regression revealed that patients with OSA had a higher odds of PE (odds ratio [OR] 3.821, *P* = 0.023), but not DVT (OR 1.971, *P* = 0.563) when accounting for significant demographic differences. Female sex and total knee arthroplasty were also associated with a higher odds of PE (OR 3.453 for sex, *P* = 0.05; OR 3.243 for surgery type, *P* = 0.041), but not DVT (OR 2.042 for sex, *P* = 0.534; OR 1.941 for surgery type, *P* = 0.565).

**Conclusion:**

Female patients with OSA may be at greater risk for VTE, specifically PE, after total knee arthroplasty. More attention toward screening procedures, perioperative monitoring protocols, and VTE prophylaxis may be warranted in populations at risk.

Obstructive sleep apnea (OSA) is a common sleep-related breathing disorder that is characterized by recurrent episodes of partial or complete obstruction of the upper airway during sleep.^1^ Advanced age, male sex, and obesity are known risk factors.^2-4^ Many studies have estimated OSA to have a prevalence between 1% and 4% in the general population.^5,6-7,8^ This prevalence is much higher in patients undergoing elective surgery, especially primary total joint arthroplasty (TJA), which has been estimated to be between 6.7% and 8.7%.^3,8,9^ However, the reported prevalence of OSA has increased over time, in part, because of the increasing rates of obesity and increasing life span, both of which are known causes of osteoarthritis and reflect the increased demand for TJA.^2,3,10-12^

Many studies have demonstrated that patients with OSA are at higher risk for perioperative and postoperative complications after TJA; however, emphasis on venous thromboembolism (VTE) has not been established.^2,4,7-9,13,14^ VTE, defined as either pulmonary embolism (PE) or deep vein thrombosis (DVT), is a rare, but serious complication and a major cause of morbidity, mortality, and healthcare costs after TJA.^15,16-17,18^ OSA is believed to be an independent risk factor for VTE because it induces rheological (ie, increasing hematocrit and blood viscosity) and systemic changes (ie, hypertension and hyperactivation of the sympathetic nervous system) that favor the genesis of thrombotic events.^19,20-21,22^ Therefore, from a pathophysiologic standpoint, patients with OSA undergoing TJA are potentially at a greater risk for VTE.

Because there is a lack of studies that investigate OSA as a risk factor for VTE in patients after TJA, the objective of this study sought to determine whether patients with a history of OSA who undergo primary TJA are at greater risk for developing VTE versus those without OSA.

## Methods

A retrospective chart analysis was done at a single academic medical institution to identify all consecutive primary TJA patients from July 2016 to November 2019 using Current Procedural Terminology (CPT) codes. The CPT code used for total hip arthroplasty (THA) was 27130, and the CPT code for total knee arthroplasty (TKA) was 27447. All patients with a history of OSA and VTE (defined as fatal or nonfatal PE alone, symptomatic DVT alone, or PE and DVT combined) were identified by the International Classification of Disease, 10th revision (ICD-10) diagnosis codes. The ICD-10 code used for OSA was G47.30, and the ICD-10 codes for VTE were I26.99 (PE) and I82.40 and I82.90 (DVT). All VTE events recorded in this study were confirmed to have occurred within a 90-day postoperative period. The method of VTE chemoprophylaxis for each patient was also recorded. Patients were nonrandomized to either 325 mg or 81 mg aspirin (American Society of Anesthesiologists [ASA]) or non-ASA therapy (ie, low–molecular-weight heparin, unfractionated heparin, direct oral anticoagulation, or warfarin). Patient baseline demographics such as age, body mass index (BMI), ASA score, sex, and race were collected as well. Study groups were separated by the primary diagnosis of OSA. Because this was a quality improvement study, it was exempt from institutional review board approval.

Patients at our institution were not routinely screened for VTE. PE was diagnosed using chest CT scans or ventilation-perfusion studies as per the current American College of Clinical Pharmacy and American Academy of Orthopedic Surgeons recommendations.^23,24^ Symptomatic DVT was diagnosed by physical examination and confirmed by lower extremity ultrasonography.

### Institutional Venous Thromboembolism Prophylaxis Protocol

From January 2016 until September 2017, patients were given 325 mg ASA to be taken orally (po) twice daily (BID) for 28 days postoperatively. After September 2017, our institution decreased this dose to 81 mg ASA po BID for patients at standard risk for VTE. Any other chemical (nonaspirin) anticoagulation agent such as enoxaparin or rivaroxaban was reserved for patients at greater risk for VTE, such as those who had a BMI over 40 or had a history of DVT or active cancer. Furthermore, any patient on an anticoagulation treatment regimen before surgery was kept on that regimen. Finally, patients received sequential pneumatic compression devices intraoperatively and postoperatively to use for at least 18 hours per day for at least 14 days.

Notably, a history of OSA was not a criterion for different VTE chemoprophylaxis such that patients with similar VTE risk profiles, regardless of whether they had a history of OSA or not, received the same regimen for VTE chemoprophylaxis.

### Statistical Analysis

All statistical analyses were performed using SPSS v25 (IBM). Descriptive data were reported as means (±SD) for continuous variables and as counts (%) for categorical data. Independent, two-tailed Student *t*-tests were performed to compare continuous data between cohorts, while chi-squared tests were used for categorical data. Chi-squared tests were conducted to compare differences in VTE rates between cohorts. A multivariate logistic regression model was also used to compare PE, DVT, and total VTE rates between cohorts to account for the notable differences in demographic data and VTE chemoprophylaxis found between the two cohorts. Type of surgery, that is, THA or TKA, was also run through the regression model to determine whether either surgery type influenced VTE rates. These findings were reported as an odds ratio (OR) with an associated 95% confidence interval (CI). The results with a *P* value of < 0.05 were considered to be statistically significant.

## Results

A total of 12,963 consecutive primary TJA patients (6,039 THA and 6,924 TKA) were identified and included in this study. Nine hundred thirty-five (7.2% rate of OSA in the study population) patients had a history of OSA, whereas the remaining number of patients did not have OSA. Chi-squared analyses revealed that total combined VTE rates were greater for the OSA group than those in the non-OSA group (6 cases [0.6%) versus 30 cases (0.25%], *P* = 0.028]. Similar trends were observed toward higher PE rates (6 cases [0.6%] versus 29 cases [0.24%], *P* = 0.023) and DVT rates (1 case [0.1%] versus 5 cases [0.04%], *P* = 0.37) in patients with OSA. When stratified by sex, a greater proportion of female patients experienced PE, DVT, and combined VTE compared with male patients (Table 1).

**Table 1:** Venous Thromboembolism Rates in Patients Undergoing Total Joint Arthroplasty With or Without a History of Sleep Apnea

Variables	Sleep Apnea (n = 935)		Control (n = 12,028)		*P* ^a^
PE rate	6 (0.6%)		29 (0.24%)		0.023
	Male	0 (0%)	Male	4 (0.03%)	
	Female	6 (0.6%)	Female	25 (0.21%)	
DVT rate	1 (0.1%)		5 (0.04%)		0.37
	Male	0 (0%)	Male	1 (0.01%)	
	Female	1 (0.1%)	Female	4 (0.03%)	
Combined VTE rate^b^	6 (0.6%)		30 (0.25%)		0.028
	Male	0 (0%)	Male	4 (0.03%)	
	Female	6 (0.6%)	Female	26 (0.22%)	

DVT = deep vein thrombosis, PE = pulmonary embolism, VTE = venous thromboembolism

a *P* values determined by two-tailed, unpaired Student *t*-test or chi-squared tests.

b Combined VTE includes subjects with both PE and/or DVT rates.

Patient demographic data were also compared between the two cohorts. On average, patients with OSA were younger than patients without OSA (64.7 ± 9.13 years versus 65.9 ± 10.7 years, *P* < 0.001), had higher BMI (35.3 ± 6.35 versus 30.5 ± 6.2, *P* < 0.001) and ASA scores (2.7 ± 0.58 versus 2.4 ± 0.6, *P* < 0.001), and were more often male (52% versus 37%, *P* < 0.001). No difference was observed in race between the two cohorts (*P* = 0.61). When classified by surgery type, 377 of 6,039 patients undergoing THA had a history of OSA, while 558 of 6,924 patients undergoing TKA had a history of OSA (6% versus 8%, *P* < 0.0001). Furthermore, a greater proportion of patients in the OSA group were placed on non-ASA therapy than patients without OSA (20% versus 13%, *P* < 0.0001) (Table 2).

**Table 2:** Patient Demographics and Characteristics

Variables	Sleep Apnea (n = 935)		Control (n = 12,028)		*P* ^a^
Age	64.7 ± 9.13		65.9 ± 10.7		<0.001
BMI	35.3 ± 6.35		30.5 ± 6.2		<0.001
ASA	2.7 ± 0.58		2.4 ± 0.6		<0.001
Sex					<0.001
Male	482 (52%)		4,282 (37%)		
Female	453 (48%)		7,746 (64%)		
Race					0.61
White	608 (65%)		7,721 (64%)		
African American (Black)	156 (17%)		1,949 (16%)		
Other race (including Asian)	171 (18%)		2,357 (20%)		
Surgery type					<0.0001
THA (n = 6,039)	377 (6%)		5,662 (94%)		
TKA (n = 6,924)	558 (8%)		6,366 (92%)		
Anticoagulation medication					<0.0001
Aspirin	678 (72%)		9,713 (81%)		
Nonaspirin therapy	193 (20%)		1,610 (13%)		
	LMWH	126 (13%)	LMWH	1,014 (8%)	
	Unfractionated heparin	2 (1%)	Unfractionated heparin	16 (0%)	
	DOAC	59 (5%)	DOAC	501 (4%)	
	Warfarin	6 (1%)	Warfarin	63 (1%)	
Unknown	74 (8%)		721 (6%)		

^a^denotes that P values determined by two-tailed, unpaired Student t-test or chi-squared tests. ASA = American Society of Anesthesiologists, BMI = body mass index, DOAC = direct oral anticoagulant, LMWH = low–molecular-weight heparin, TKA = total knee arthroplasty

To account for these differences between the two cohorts, multivariate logistic regressions were used to measure the association between OSA history and PE, DVT, and total VTE rates (Table 3). When looking at a history of OSA alone, the model predicted that patients with OSA have a significantly higher odds of acquiring PE {OR 2.672 (95% CI: [1.107 to 6.453]), *P* = 0.029} and VTE {OR 2.583 (95% CI: [1.072 to 6.221]), *P* = 0.034}. A higher odds of having DVT was also observed in patients with OSA {OR 2.575 (95% CI: [0.3 to 22.059])}; however, these results were not statistically significant (*P* = 0.388). When accounting for a history of OSA and the significant differences seen in our demographic data (eg, age, BMI, ASA score, and sex) and anticoagulation medication, patients with a history of OSA were still predicted to have a significantly increased odds of having a PE {OR 3.821 (95% CI: [1.206 to 12.107]) *P* = 0.023} and combined VTE {OR 3.594 (95% CI: [1.144 to 11.284]), *P* = 0.028}. Again, there was an increased odds of having DVT in patients with OSA {OR 1.971 (95% CI: [0.197 to 19.705])}; however, these results were not statistically significant (*P* = 0.563). Female sex with or without OSA was also associated with a higher odds of PE {OR 3.453 (95% CI: [1.001 to 11.910]), *P* = 0.050} and combined VTE {OR 3.600 (95% CI: [1.050 to 12.346]), *P* = 0.042} and trended toward higher odds of DVT, although not significant {OR 2.042 (95% CI: [0.215 to 19.392]), *P* = 0.534}. Furthermore, patients undergoing TKA with or without OSA were found to have a higher odds of PE {OR 3.243 (95% CI: [1.052 to 9.994]), *P* = 0.041} and combined VTE {OR 3.392 (95% CI: [1.111 to 10.357]), *P* = 0.032}, but not DVT {OR 1.941 (95% CI: [0.202 to 18.632]), *P* = 0.565}. Other demographic data, such as age, BMI, ASA score, and anticoagulation therapy with or without OSA, did not increase the risk of VTE outcomes.

**Table 3:** Multivariate Logistic Regression for Venous Thromboembolism Rates

Variable	Unadjusted Model						Adjusted Model					
	PE (95% CI)	*P*-value	DVT (95% CI)	*P*-value	VTE (95% CI)	*P*-value	PE (95% CI)	*P*	DVT (95% CI)	*P*	VTE (95% CI)	*P*-value
Sleep apnea^a^	2.672 (1.107-6.453)	0.029	2.575 (0.3-22.059)	0.388	2.583 (1.072-6.221)	0.034	3.821 (1.206-12.107)	0.023	1.971 (0.197-19.705)	0.563	3.594 (1.144-11.284)	0.028
Sex^b^	—	—	—	—	—	—	3.453 (1.001-11.910)	0.050	2.042 (0.215-19.392)	0.534	3.600 (1.050-12.346)	0.042
Surgery type^c^	—	—	—	—	—	—	3.243 (1.052-9.994)	0.041	1.941 (0.202-18.632)	0.565	3.392 (1.111-10.357)	0.032

ASA = American Society of Anesthesiologists, BMI = body mass index, DVT = deep vein thrombosis, PE = pulmonary embolism, VTE = venous thromboembolism

a Control group was used as reference.

b Male sex was used as reference.

c Total hip arthroplasty (THA) was used as reference.

Unadjusted model: VTE = constant + sleep apnea.

Adjusted model: VTE = constant + sleep apnea + age + BMI + ASA + gender + anticoagulation medication + surgery type.

## Discussion

The results of our study suggest that OSA is an independent risk factor for increased PE and combined VTE rates, but not DVT. A multivariate logistic regression model isolating OSA's effect revealed that OSA markedly increases the odds of PE and combined VTE in patients undergoing primary TJA by twofold. These findings are further corroborated by the results of numerous studies that have highlighted the increased risk of postoperative pulmonary complications in surgical patients with OSA.^2,9,14,25-27^ Vakharia et al conducted a retrospective review from 2005 to 2014 using Medicare Standard Analytical Files to compare 90-day complications in demographically matched patients with or without OSA undergoing primary TJA. Like our study results, they found that patients with OSA had a greater odds of developing PE after TKA {OR 1.51 (95% CI: [1.11 to 2.04]), *P* = 0.007}, but not THA {OR 1.30 (95% CI: [0.78 to 2.17]), *P* = 0.303}.^2^ Similarly, Naqvi et al^14^ studied a total of 4,984 patients with and without OSA after TJA who were matched using a 1:3 ratio and reported an increased rate of pulmonary complications, which included PE, in patients with OSA {1.7% versus 0.6%, OR 2.91 (95% CI: [1.58 to 5.36]), *P* < 0.001}. Furthermore, D'Apuzzo and Browne^9^ reviewed a National Inpatient Sample database from 2006 to 2008 comparing in-hospital postoperative complications in a total of 258,455 patients with or without OSA undergoing revision TJA and determined that OSA was associated with increased PE (OR 2.1, *P* = 0.001). Even when accounting for any demographic differences between the two groups, including age, BMI, and ASA score, our model still found a significant threefold increase in odds of having PE in patients with a history of OSA alone (*P* = 0.028). These odds are also independently increased threefold in female patients (*P* = 0.042) and threefold in patients undergoing TKA (*P* = 0.032). Although previous studies have identified a higher prevalence of OSA and severity in males than in females, other studies argue that female patients are less frequently diagnosed and treated for OSA than male patients.^2-4,28^ More importantly, these studies report that the consequences of the disease are at least the same in female patients, if not worse for comparable degrees of severity.^28^ This suggests that female patients presenting with OSA, despite other risk factors, have a clinically notable increased risk of having a PE after TKA and may require greater attention toward management and treatment.

Although multiple studies concur that patients with OSA undergoing TJA experience greater pulmonary complications than patients without OSA, some studies did not report such findings.^7,8,13^ Berend et al^7^ retrospectively reviewed 1,255 consecutive patients undergoing TJA at a single hospital between 2005 and 2006 and found no in-hospital DVT or PE in any of their patients with or without OSA. One possible explanation for this finding, aside from the fact that in-hospital VTE is a rare occurrence in itself, could be due to the implementation of their institution's postoperative OSA protocol, which they credit to have lowered their rate of notable in-hospital complications. This protocol includes continuous pulse oximetry monitoring 24/7 until discharge, immediate use of continuous positive airway pressure or bilevel positive airway pressure in the postoperative period, cardiac monitoring/telemetry for 24 hours after arrival to in-patient unit, promotion of rapid advancement to oral pain medications with avoidance of narcotic drugs if possible, head of bed elevation to 30° to 40° if not contraindicated, and OSA educational material distribution to patients before hospital discharge.^7^ Moreover, our study does not solely focus on in-hospital VTE rates, but rather VTE events that occur within a 90-day postoperative period. Parikh et al^8^ studied a small cohort of 19 TJA patients with a preoperative diagnosis of moderate or severe OSA and did not report any VTE events in their patients. Although they did report serious postoperative pulmonary complications such as respiratory arrest and respiratory depression, they only reported a small number of cases and within a 3-day postoperative phase in hospital. Thus, it is likely that they did not detect any VTE events because of the size and length of scope of their study and its design. Cashman et al^13^ identified TJA patients with OSA between 1998 and 2008 at a single institution and found no differences between OSA and control patients in cardiovascular and respiratory complications after TJA. However, the authors gave a broad definition of what they considered as a respiratory complication, which does not entirely encompass the scope of VTE to the degree that our study does. In addition, like Berend et al, they credit their institution's postoperative monitoring protocols to successfully reduce postoperative complications commonly caused by OSA, which may include VTE.

The need for screening procedures and perioperative and/or postoperative interventions to identify undiagnosed OSA and minimize VTE risk in patients undergoing TJA is the main concern for practicing arthroplasty surgeons. Harrison et al^4^ reported the incidence rate of undiagnosed OSA in patients scheduled for elective TJA at their institution to be 4%. This literature identifies advanced age, male sex, and obesity or high BMI as risk factors and strong predictors of OSA.^2-4^ In concordance with previous studies, patients with OSA reported in our study were markedly more likely to be male with higher BMI and ASA. Preoperative questionnaires such as the Berlin Questionnaire, ASA checklist, and the STOP-BANG (Snoring, Tiredness during daytime, Observed apnea, high blood Pressure, BMI, Age, Neck circumference, and Gender) questionnaire have been used to identify OSA in the surgical population and are believed to prevent perioperative complications in patients at risk.^3,4^ In fact, patients undergoing elective surgery at our institution are identified with OSA using the STOP-BANG questionnaire during preoperative evaluation by anesthesiology. Other preoperative methods of identifying surgical patients who are at risk of OSA that have also been implemented include nocturnal pulse oximetry and home sleep testing. Intraoperative management for patients with OSA mainly focuses on surgical measures and type of anesthesia. Orthopedic surgeons should seek to minimize the surgical stress and duration of surgery because both factors have been shown to increase perioperative complications.^3^ Moreover, regional or local anesthesia is preferred in patients with OSA because it has been shown to reduce rates of pulmonary and gastrointestinal complications, acute anemia, and mortality when compared with OSA patients using general anesthesia.^3,14^ It is suggested that patients with OSA who undergo general anesthesia should receive greater attention and monitoring in the postoperative period to ensure upper-airway patency.^3,4^ In the postoperative period, hospital systems should encourage the use of continuous positive airway pressure, continuous use of pulse oxygenation with pulse oximetry, and avoidance of opioids and sedative drugs for pain control.^2,3,8^ Utilization of these protocols has been shown to reduce the many perioperative and postoperative complications that patients with OSA experience, including those related to VTE.

Finally, the use of VTE prophylaxis in patients with OSA may require greater attention. The current American College of Clinical Pharmacy and American Academy of Orthopedic Surgeons guidelines recommend the use of pharmacologic agents (grade 1B recommendation) and/or mechanical devices (grade 1C) in patients undergoing elective THA or TKA who are not at elevated risk for VTE or bleeding beyond the surgery itself.^23,24^ For most patients undergoing TJA, the use of low–molecular-weight heparin or direct oral anticoagulant, such as rivaroxaban or apixaban, is suggested in preference to the other recommended agents (grade 2B).^23,29^ The guidelines at our institution reserve the use of these agents for patients at high risk of VTE including those with a BMI over 40 that may also be at risk of OSA because low-dose aspirin has been shown to be safe and effective for VTE prophylaxis in patients at standard risk of VTE.^29,30^ With our study's current findings, it would not be unreasonable to classify female patients with OSA undergoing TKA at high or moderate risk for VTE and thus recommend consideration of stronger VTE chemoprophylaxis agents. For instance, although a greater percentage of patients with OSA in our study were treated with stronger VTE chemoprophylaxis agents than patients without OSA (20% non-ASA therapy in the OSA group versus 13% non-ASA therapy in the control group, *P* < 0.0001), our study still found higher VTE rates in patients with OSA, which strengthens our findings in support for more aggressive VTE treatment in patients with OSA. A subanalysis including only patients at standard risk for VTE prescribed ASA because VTE chemoprophylaxis revealed the exact same trends as this study (ie, higher PE rates in female patients with OSA undergoing TKA), which further increases the validity of these findings. However, we cannot comment on which nonaspirin anticoagulant agent would be the most optimal nor can we recommend how aggressively surgeons should escalate chemoprophylaxis therapy based on these data alone. Future studies may wish to investigate the optimal VTE prophylaxis treatment regimen and risk stratification protocol for this unique population.

The limitations of this study are mainly inherent to its retrospective study design which increases the susceptibility of our data to selection bias that may have contributed to the differences in demographic data seen in our study population. In response, we ran a multivariate logistic regression to study their effect on our reported VTE rate and found no such correlation. Moreover, because our retrospective data collection spans over 3 years and only includes a cohort of prospective, consecutive TJA cases, we believe that these factors may further limit the possible selection bias that may influence the results of our study. Future studies, however, may wish to conduct a prospective approach to confirm these results, especially for DVT rates. Although our study's retrospective period of 3 years may be considered a strength, this long study period may also expose our study to confounding variables such as institutional changes and deviations in our institution's VTE prophylaxis guidelines, which may bias the results of our reported VTE rates in patients with OSA. Future studies may wish to investigate the effect of different VTE prophylaxis protocol changes on VTE rates in patients with OSA to confirm this limitation. Finally, we acknowledge that our results show very low VTE rates (ie, ≤30 total cases), and therefore very small differences, which call into question our study's statistical and clinical relevance. However, our reported rates follow the decreasing trend of VTE rates as reported in the orthopedic literature. These low rates are likely due to a combination of factors such as improvements in surgical technique and perioperative care, faster postoperative mobilization protocols, shorter duration of hospitalization, and more consistent use and/or longer duration of VTE prophylaxis.^31^ Although our small numbers may suggest questionable statistical and clinical relevance, it is still important to identify independent factors that increase the risk of VTE to minimize morbidity and mortality in patients undergoing TJA. To the best of our best knowledge, our study is the first to identify OSA as an independent risk factor for VTE in combination with female sex and TKA procedures. This provides important information relevant to practicing orthopaedic surgeons to heighten awareness of patients presenting with OSA, especially those who are female and undergoing TKA, as a risk category for VTE disease.

## Conclusion

The findings of our study identify a trend that a history of OSA markedly increases the odds of a patient having a PE after their total joint arthroplasty surgery, especially in female patients undergoing TKA. Greater attention toward screening procedures, perioperative and postoperative monitoring protocols, and VTE prophylaxis may be warranted in populations at risk to prevent future complications.
